# Smart Accessibility and Quality of Life in Education: A Systematic Review of Adolescent Support and Universal Access

**DOI:** 10.7759/cureus.79494

**Published:** 2025-02-23

**Authors:** Naeema Abdulrahman Alhasan, Marco Lombardi, Nasser Saad Al-Ajmi

**Affiliations:** 1 Department of Special Education, College of Education, King Saud University, Riyadh, SAU; 2 Department of Social Education Care Work and Equality Research, Hogent University, Ghent, BEL

**Keywords:** quality education, quality of life, quality of life (qol), sdg 4, smart technology

## Abstract

The accelerated digitalization of secondary education following the COVID-19 pandemic has fundamentally altered the nature of learner engagement and participation with various knowledge resources. From digital lessons to gamification to integrated smart technologies capable of supporting specific learner needs, the opportunity for inclusion and universal accessibility is unprecedented. This study represents an important extension of prior research in this field, combining multiple empirical studies regarding smart technologies, accessibility effects, and learner quality of life (QoL) into a blueprint for future educational applications. Through a systematic literature review (SLR), studies from multiple databases using multiple empirical methods have been identified and thematically compared. The findings reveal that while smart technologies have the potential to revolutionize inclusive education, accessibility gaps persist, particularly for students with special needs, leading to disparities in learning opportunities and outcomes. Such gaps stem from insufficiently adaptive technologies, inadequate teacher training, and limited resource allocation for underserved communities. By targeting a bottom-up, participative design approach to technological identification and integration, a broader range of student needs can be accommodated, and technological accessibility can be ensured for a larger percentage of the secondary student population. This study recommends improving educational outcomes for all students, especially those with special needs, by prioritizing the development of adaptable, inclusive technologies and continuous utility assessments. This research synthesizes findings from multiple studies to evaluate the impact of smart accessibility on adolescent learning and quality of life, providing a framework for assessing and improving technological integration in secondary education.

## Introduction and background

Following the disruptive effects of COVID-19 on global educational practices, digitalization emerged as both a viable long-term solution to changing learner needs and a practical solution for various considerations related to student accessibility and accommodation [1]. In educational settings, smart technologies are predicted to improve learning equity, ensuring the accessibility of learning resources, opportunities, and accommodations while providing students with alternative pathways to improve their self-directed learning outcomes [2]. From traditional learning supplements like digital lessons, gamification, or peer chatrooms to specialized solutions for special needs students, the digitalization of education via smart technologies presents remarkable opportunities for accessibility and inclusion [3]. Within this context of technology-enabled learning, SMART (Self-directed, Motivated, Adaptive, Resource Enriched, Technology-Embedded) technologies and assistive resources are being developed to support students across varied learning abilities and competency levels [4].

Quality of life (QoL) is defined as complete social, mental, and physical well-being involving a multidimensional concept with physical and psychosocial aspects [5]. QoL includes various domains of well-being such as interpersonal relations, rights, social inclusion, emotional well-being, physical well-being, and material well-being [6]. Moreover, according to one more study, several domains have particular implications for educational systems, such as self-determination and social inclusion, which predict the productive effects of systemic resources, student support, and learner attainment over their educational lifecycle [7].

Self-determination in educational settings is an important antecedent to inclusion, whereby it empowers students to make decisions and choices for their education that underlie their strengths, desires, and interests [8]. Furthermore, specific student protections have been used to promote self-determination, including equal opportunity, full participation, independent living, and goal-setting and -developing skills [9].

Brock et al. (2020) observe that educational systems can develop support and accommodation arrangements by targeting the relationship between QoL, self-determination, and agency [6]. This study offers practical insights into techno-institutional solutions that can be applied to a student-centered learning solution based upon emergent technologies, accessible digital pedagogy, and learner accommodation in secondary education [7].

Ioana-Alexandra et al. (2021) emphasize the importance of accessibility in resource design, focusing on the functional effectiveness of technologies for individuals with special needs [10]. They suggest integrating smart technologies like IoT (Internet of Things) into educational systems to monitor student needs, provide support, and signal intervention needs based on ongoing outcomes [9].

Central to this interpretation of accessibility, accommodation is the core concept of service quality. In educational settings, improvement in service quality could be achieved through several strategies, including professional development, regular feedback, collaboration with other educators, and decision-making by researching improvements to make. Vincent-Lancrin (2022) predicts variables related to teacher competencies, technological capabilities, learner skills, technological proficiency, and resource availability that challenge such technologies' efficacy and universal value [2]. By incorporating smart devices, including mobile phones, tablets, and accessories, into educational settings, Goksu et al. (2016) suggest that innovative software solutions can be used to resolve systemic gaps and learning disadvantages among special needs and disadvantaged groups of learners [3].

Moreover, Lombardi et al. (2016) analyzed a model in which clients’ desires, support needs, desires, environmental factors, goals, and support strategies combined to predict the quality of life outcomes [10]. According to a study, 26.7% of the quality of life outcomes changes have been found [11]. To meet each person's unique needs and ensure they have the resources they need for success and well-being, it is essential to understand their specific support needs. Data from numerous users can be analyzed to improve resource allocation and overall system efficacy by identifying common trends and regions with high demand [12]. The support paradigm has been merged with the quality of life to construct the Quality of Life Support Model (QOLSM). For instance, to enhance the ability to participate in community and social activities, people with intellectual disability could be provided with assistive technology and communication devices. This will also help them overcome communication barriers [13].

This research aims to critically analyze the concepts and operative solutions surrounding smart accessibility in secondary education, weighing the findings from multiple empirical studies to highlight the specific domains and variables needed to frame inclusive improvements in future educational systems. Through a systematic review, it aims to: define and critique the concept of accessibility and identify the emergent role of smart technologies in an educational context; evaluate educational inclusion from the perspective of special needs learners; assess the relationship between accessibility and accommodation in recent changes to educational systems (e.g., hybrid learning) and identify opportunities for smart technology enhancement in the future; propose a blueprint of accessibility and inclusion through the inclusion of smart technologies; incorporate a support needs model to enhance the quality of life in special needs or intellectually disabled students.

## Review

Methodology

Research Approach

The current research has adopted the systematic literature review (SLR) techniques described in prior studies regarding academic innovations and inclusion, such as Brock et al. (2020), to weigh the comparative findings of multiple empirical studies [6]. The central problem explored throughout this SLR was the relationship between the accessibility needs of students in secondary education and the possible role of smart learning technologies in enhancing and sustaining positive QoL effects.

Quality of life is a complex and multifaceted concept that varies according to the scope, perspective, and criteria applied to its assessment. However, QoL in educational settings derives from an outcome-oriented viewpoint, which focuses on the interventions and overall impact on the well-being and satisfaction of individuals [14]. To formalize QoL research and clinical assessments, Schalock (1996) identified eight core domains demonstrated in Table 1 [13].

**Table 1:** General QoL indicators QoL: quality of life

Dimension	Domains	Indicators
Emotional Well-being	Safety, spirituality, happiness	Freedom from stress, self-concept, contentment
Material Well-being	Ownership, financial security, food reliability	Employment, possessions, socioeconomic status, shelter
Physical Well-being	Health, nutrition, recreation mobility	Healthcare, leisure, active living
Self-Determination	Autonomy, choices, decisions	Personal control, self-direction, personal goals/values
Personal Development	Education, skills, fulfillment	Personal competence, purposeful activity, advancement
Social Inclusion	Acceptance, status supports, roles	Community activities, work environment, volunteering, residential environment
Interpersonal Relationships	Intimacy, affection, family	Interactions, friendships, support
Rights	Privacy, access, voting	Due process, ownership, civic responsibilities

**Figure 1:**
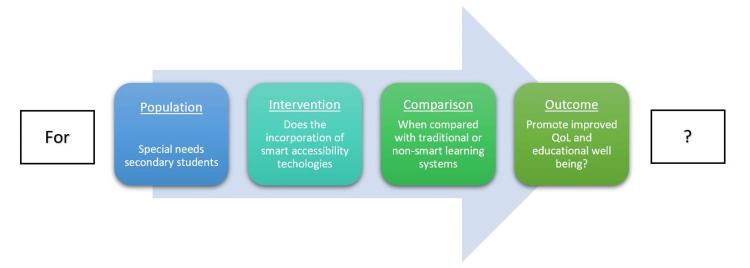
PICO model of search question PICO: Population, Intervention, Comparison, and Outcome Image credits: Authors

The following PICO (Population, Intervention, Comparison, and Outcome) instrument (Figure 1) was developed to hypothesize and contextualize the targeted literature for this review. Within this query is the central target population or those students with special needs who are candidates for secondary educational accessibility interventions. It is hypothesized that student learning experiences will be enhanced, quality of life will be improved, and over time, self-determination effects will mature and improve educational performance. Furthermore, the research is reviewed independently by two researchers.

Search Strategy

For this study, the keyword development process involved performing multiple search functions until the identified range of studies was complementary to the core problem and its underlying variables of QoL, self-determination, smart technology, and accessibility. The details of the search terms' variations and the associated Boolean modifiers that were used to perform these searches from multiple databases regarding this multidimensional research problem are compiled in the table in Appendix A. Due to the variations in prior research, each of these searches was performed to identify studies meeting the prescribed conditions of the study.

Inclusion and Exclusion Criteria

Most importantly, by narrowing the scope of research to focus only on education-based accessibility issues, empirical research, and smart technologies, this study has established a clear and relevant sample population and support modality. In administering these criteria, it was found that the scope of available studies across three popular databases (ScienceDirect, Taylor & Francis, and Sage) along with PubMed, Scopus, Web of Science, Google Scholar, and IEEE Xplore) was narrowed to reflect a specific subset of highly insightful and problem-specific studies. Table 2 shows the inclusion-exclusion criteria for the study.

**Table 2:** Inclusion-exclusion criteria

Inclusion	Exclusion
After 2015	Before 2015
Focused on Education Accessibility	Focused on Broader Accessibility Considerations
Smart Technology	General Technology Concerns
Special Needs Students	Other General Student Considerations
Quality of Life Considerations	Other Factors, Such as Residency or Transportation
Secondary Education	Other Educational Levels
Primary Research	Secondary Research
Credible/ peer-reviewed articles	Non-peer-reviewed/ non-credible
Full text available	Full text not available

Source Selection and Outcomes

**Figure 2:**
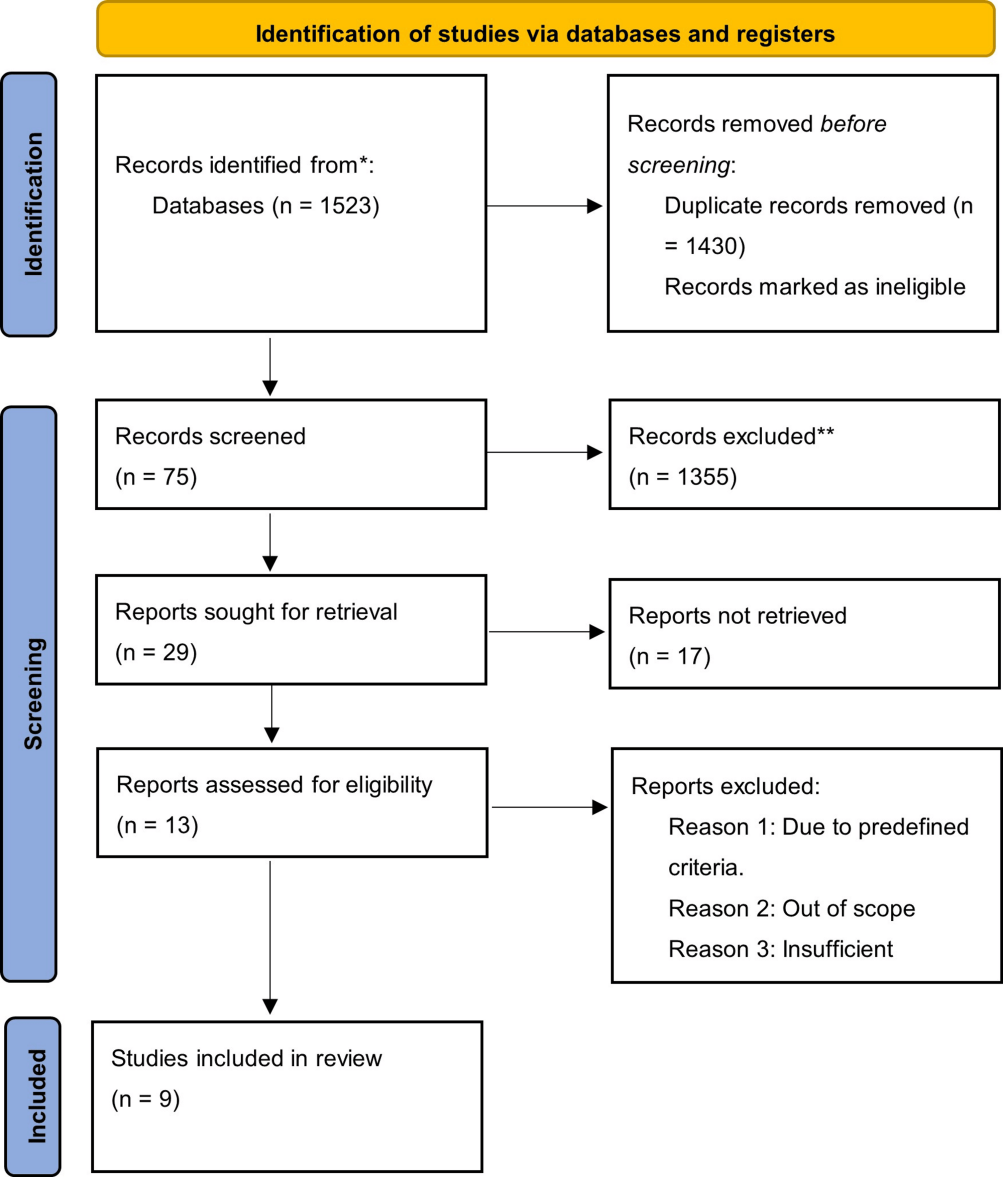
PRISMA flow diagram PRISMA: Preferred Reporting Items for Systematic Reviews and Meta-Analyses

In SLRs, the Preferred Reporting Items for Systematic Reviews and Meta-Analyses (PRISMA) guideline was developed to increase quality and transparency in reporting the systematic review by describing a minimum set of characteristics that were applied to the funneling and selection of integrated studies [15]. Figure 2 presents the PRISMA flow diagram that was used to collect and weigh the eligibility of these studies. Perhaps the most critical impact variables were the emphasis on secondary education, empirical results, QoL-based indicators, and smart technological innovations. The resultant cohort of studies was robust, comparable, and anchored to the underlying concepts and theories that initiated this study.

A modified Critical Appraisal Skills Programme (CASP; 2023) appraisal instrument was developed to assess the quality and reliability of these studies (Appendix B). Each study scored level 1-3 on 10 overall categories. A score of 10 indicated an exemplary, reliable study, with any studies receiving a score greater than 20 excluded from the end sample. The resultant sample was subsequently analyzed for critical insights related to the core findings, underlying themes, and any focus-based evidence. Appendix C provides the results of the SLR output matrix, which details three primary elements, including the methodology, findings, and implications of each study regarding smart technologies and educational accessibility. Appendix D presents the thematic analysis of the studies, detailing the core significance of each investigation and the focus of this study.

Data Analysis and Interpretation

One of the challenges in measuring variables related to both accommodation and QoL is that their evaluation in both theory and practice is varied and often inconsistent across studies. Harrison et al. (2020) suggest that dividing overlapping themes into centralized ones, like well-being and social functioning, can help balance the over-diversification of measures and domains [15]. Key thematic elements related to QoL-related outcomes were extracted from their findings. From a general perspective, techno-functional assessment is based upon a critical comparison of experiential effects and smart interfaces on student learning outcomes concerning the broader classroom median and the individual’s arc of development or achievement [16]. Therefore, consideration was given to this form of a cause-effect relationship, whereby smart technologies were critically assessed with their impacts on accessibility and accommodation-based roles in secondary education.

Results

SLR Data Extraction

The source identification process for a study on smart technologies in education focuses on their accessibility and QoL effects. Based on foundational studies, the research aims to identify proxy elements from these studies to focus on practical lessons for future integration. The findings highlight the significance of smart technology inclusion in learning, achievement, behavioral adaptation, and self-determination and their implications for future technology applications. The table in Appendix C shows the SLR matrix in summarized form.

Digitalization and Technology Effects in Secondary Classrooms

As schools explore new digital capabilities, the array of new applications and technologies has resulted in pedagogical confusion and undermined the goal-oriented effectiveness of advanced technologies. Starks and Reich (2023) observe that due to smart technologies like AI and interactive applications, special needs students are encouraged to assimilate into general education services or excluded from these technologies. Factors like time, digital literacy, computing resources, and classroom accessibility impose additional burdens, leading to unequal learning opportunities [17]. Miguel-Revilla et al. (2021) suggest that students assume a greater role in shaping their learning process and knowledge outcomes through techno-enhanced self-directed learning [17]. Ultimately, the institutional recognition of digital opportunities and techno-pedagogical solutions predicts the transition from traditional to hybridized classroom environments [18].

The technological advancement identified in these studies is the gamification of education, a subject addressed by Gutierrez et al. (2023) [18], and digital learning technologies by Yang et. Al (2023) [19], particularly for students with special needs or who lack the digital skills to navigate new platforms. However, the game-specific design of various in-class resources was found to have limited, if any, educational value for students of varying levels [19,20]. Moreover, a study has demonstrated that student usage and motivated engagement in such applications depend on their personal and functional value. Text-to-speech applications that assist visually impaired learners will have significantly greater long-term benefits than general interactive games or learning adventures with limited practical purposes [21].

Accessibility and the proposition of smart inclusivity: Research by Loveys and Butler (2023) shows that assistive technologies that cater to students' varying needs are crucial for productive learning outcomes [22]. The example of Nordstrom et al. (2019) associates productive learning outcomes with assistive technologies designed for students with varied needs. However, these accommodations are often absent in most secondary schools and restricted by technological adoption and deployment [20]. Starks and Reich (2023) define smart technologies as those adaptable over time to the changing needs of teachers, classrooms, and students [17]. Gamification, for example, is a potentially valuable technological innovation but is criticized for its limited transferability and adaptability across platforms [17]. Nordstrom et al. (2019) highlight not only the pragmatic value of assistive applications in supporting students with special needs but also highlight challenges associated with teacher usage, student skills, and application adaptability [20]. The interaction between need, functionality, and skill sets determines assistive technology's net value and long-term usability in secondary education scenarios [21].

QoL Life opportunities and hurdles: Starks and Reich (2023) argue that while smart technologies may seem useful for improving student learning outcomes, their accessibility to special needs students can lead to developmental and skills-based barriers, potentially delaying learning and increasing learner gaps compared to peers [17]. Assistive technologies, by virtue of their design, are supposed to provide teachers with solutions that can be used to improve student learning outcomes and enhance overall classroom experiences [23].

SLR Thematic Findings

Throughout each of the studies identified in this SLR, primary themes were extracted based on a structured thematic analysis approach. This process involved the following four aspects.

1. Initial screening and categorization: Relevant studies were reviewed to identify recurring concepts and discussion points related to digital learning, accessibility, and student experiences.

2. Coding and theme clustering: Key terms, patterns, and relationships were coded and grouped into broader thematic categories. The most frequently discussed and conceptually significant aspects were classified under Core Themes (major topics of focus), Impact Factors (influences on student experiences), and Mediators (variables that shape learning outcomes).

3. Weighting and prioritization: Thematic importance was determined by evaluating the prevalence of these themes across studies. This was done by assessing citation frequency, direct mentions, and contextual significance within the literature.

4. Visual representation: To better illustrate these findings, word clouds were generated, visually representing the prominence of different thematic elements across the reviewed studies.

Core Themes

**Figure 3:**
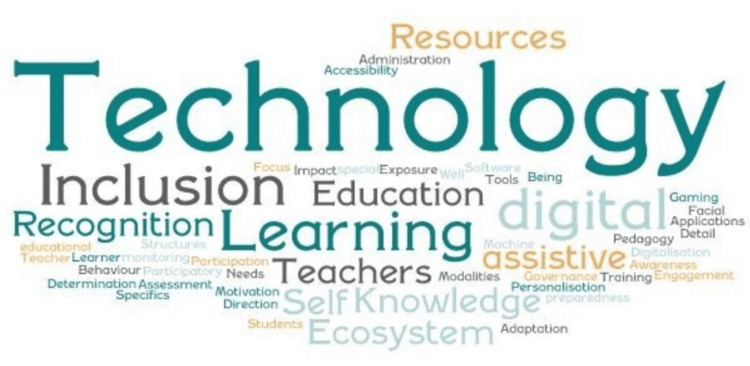
Core theme Image credits: Authors

As visualized in Figure 3, at the highest level of interpretation, the core themes identified across studies included general technological applications in education, inclusion considerations, learning effects, and key mediating variables such as teacher involvement, assistive technologies, educational applications, and recognition of learning opportunities.

Focusing on the instrumental effects directing innovative technologies in secondary education, Gutierrez et al. (2023) argued that teacher engagement and attitudinal support for digital learning and gamification are critical for successfully integrating technology into secondary education [18]. Moreover, facial recognition technologies might add value in attention-tracking, behavior-monitoring, or profile recognition; however, some students may not be able to use these functions, and some classrooms may lack the resources to integrate such functionality into their learning systems. Therefore, when developing integrative solutions, accessibility becomes more than just a special needs case solution; instead, it is a functional predictor of the tangible benefits of technology for the students and teachers over the educational lifecycle [18,22].

Impact Factors

**Figure 4:**
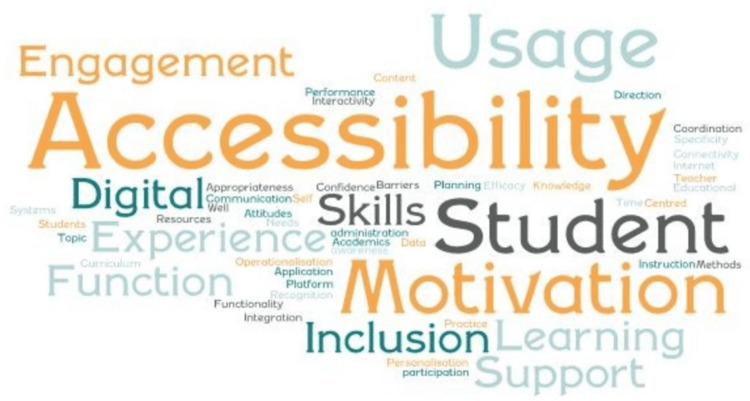
Impact factors Image credits: Authors

If technological solutions are going to have a positive effect on student learning outcomes, various impact factors must be considered. Synthesizing the findings from these studies into their core themes, Figure 4 highlights several prominent antecedents to technological effectiveness, including accessibility, usage, student engagement, and motivation.

The rapid digitization of educational resources has led to significant accessibility issues for diverse students, influenced by ideological bias, data governance, and generalization. The interactive nature of technology can obscure learner gaps, restricting independent thinking and exploration of complex concepts. Arslan-Ari and Baser (2023) suggest that teachers need specialized training and practical experience with various classroom resources and assistive technologies to improve their adaptation in future teaching experiences. This training should focus on conceptual and practical learning to ensure teachers are comfortable with the classroom applications of new technologies [23].

Mediators and QoL

**Figure 5:**
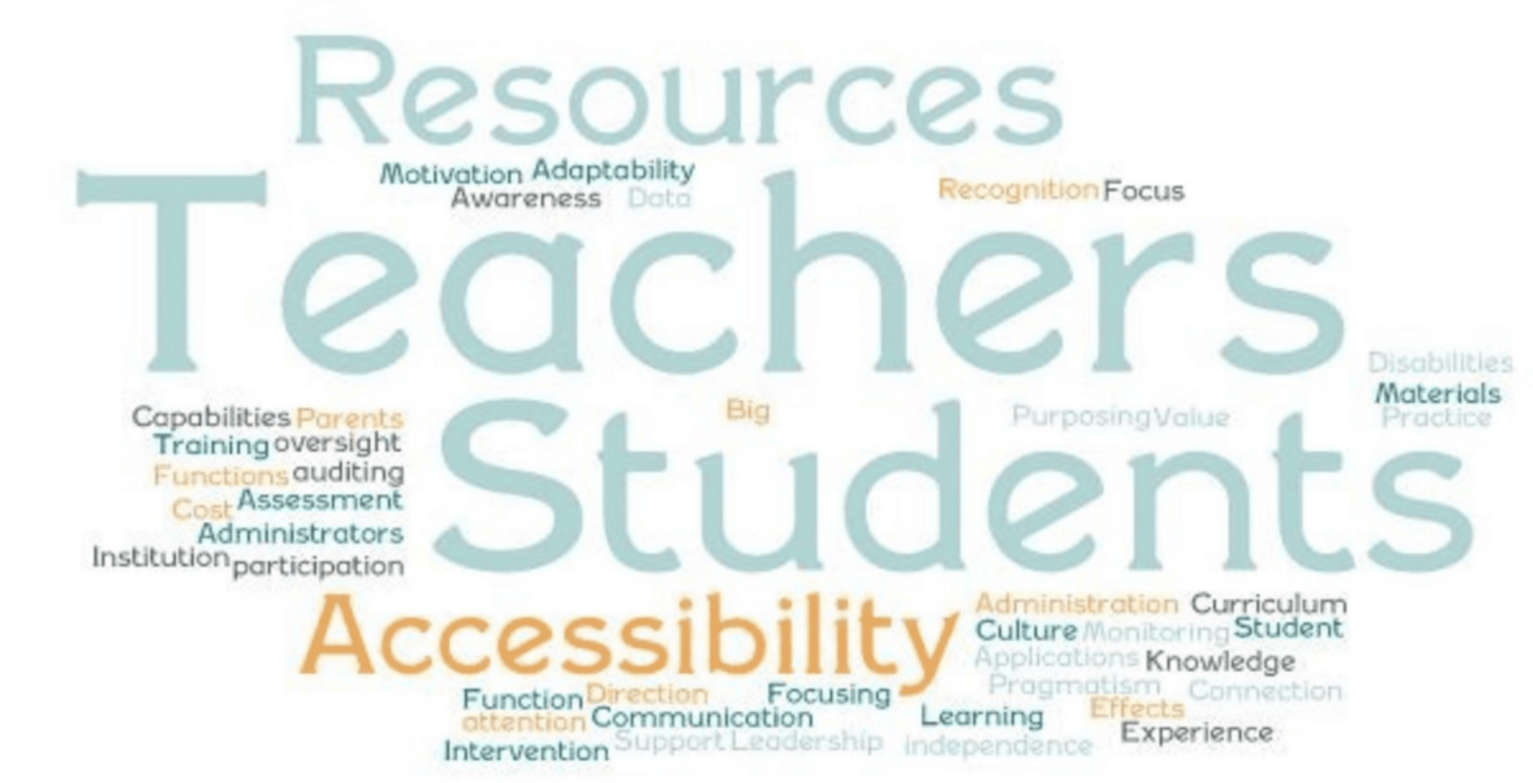
Mediators and QoL Image credits: Authors

The final series of themes visualized in Figure 5 focused on those mediating variables most likely to impact student QoL regarding learning and developmental outcomes, extend interdependent and complementary, producing effects extending throughout including teachers, students, and resources. The findings from these studies show that smart technologies must be accessible to students in terms of availability, function, and utility to enhance QoL outcomes lifecycle. The conclusions of these studies show that smart technologies must be accessible to students in terms of availability, function, and utility to enhance QoL outcomes. They must also be adaptable according to the student's distinct needs, which will likely change over time according to student and teacher usage patterns.

For smart technologies to be effective, these studies universally confirmed that teachers must receive adequate and specific training according to the software, applications, and resources used in the classroom. This means that where Yang et al. (2023) view administrative oversight as the central foundation of data selection and democratization, teacher involvement in planning and administering course applications is essential to supporting the realization of productive classroom outcomes [19]. Furthering this pragmatic value of teacher training and preparedness, Miguel-Revilla et al. (2021) introduced the concept of integrated teacher and student training to inform digital literacy as an antecedent to knowledge acquisition [17]. The relationship is simplified to a direct impact factor, whereby a deeper understanding of technology capabilities and expectations fosters deeper engagement and enhances student motivation during lesson navigation [18]. Suppose both partners in this educational arrangement attain the skills and capabilities needed to fulfill specific developmental goals. In that case, the effect on learner QoL will be magnified according to self-determination, agency, and social engagement effects.

Kumm et al. (2021) demonstrated how real-time monitoring of student attention and lesson participation can reorient and reinforce behavioral outcomes and enhance student motivation and attentiveness. The underlying QoL benefits of such technologies extended beyond the accessibility benefits of classroom support. They included a self-determination benefit associated with improved learner self-monitoring and decision-making confidence [24]. As exemplified by Miguel-Revilla et al. (2021), when students gain agency by self-moderating and self-managing their digital learning process, the productivity of the effects for learner confidence and self-efficacy is increased, potentially enhancing positive learning outcomes in the future [17].

Model of Smart Technology Accessibility for Secondary Education

**Figure 6:**
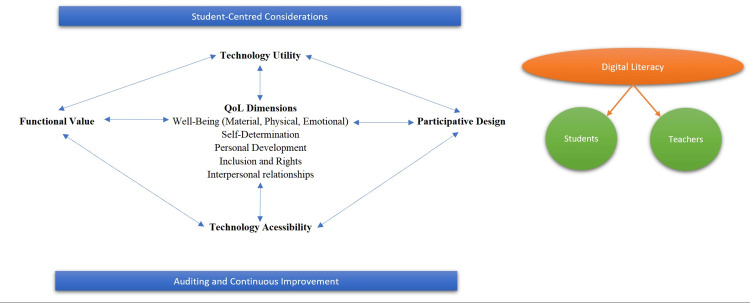
Diamond model of smart technology accessibility for secondary education Image credits: Authors

The output of this integrated review of empirical research regarding technology accessibility, student accommodation, smart solutions, and educational digitalization is a model that can be applied to secondary education in the future. Figure 6 synthesizes this evidence into a diamond model of technology accessibility buttressed by two critical domains, including student-centered considerations, auditing technologies, and continuous improvement of classroom offerings. The following describes each of these quadrants relative to their role in optimizing the practical benefits of smart technologies in the future lifecycle of all secondary students, particularly those with special needs.

Diamond Model

Functional value: Characterized by both lesson-specific effects and learner outcomes, applications' functional value is a core antecedent to their positive impact on student well-being, particularly self-determination and personal development [24].

Technology utility: Although many new technologies are being introduced into classroom settings, the inclusive utility of these technologies plays an important role in their universal value for educational settings [18].

Technology accessibility: If the utility of the technology is functional and broadly defined, it must be accessible to all students. This means that accommodation should be considered for students with special needs, including additional modules, app modifications (e.g., zoom, text-to-speech), or sensor-based technologies [22].

Benchmarks

Student-centered considerations: Bottom-up design prioritizes student benefits, focusing on technologies that are not simply replacements for traditional classroom resources but are transforming the educational landscape in a productive and valuable way. Special needs students require special considerations such as incorporating accessibility and accommodation into a full-spectrum QoL solution.

Auditing/continuous improvement: Technological functionality must be verified through regular audits and an institutional commitment to continuous improvement that aligns student performance outcomes with module or instrument objectives [19,20].

Mediators

Digital literacy: There is an expectation of uniform digital literacy in many schools that excludes student populations with specific needs or disabilities from participation in a growing number of technology-supported resources and applications [17]. Digital literacy was universally identified in these studies as a core antecedent to the functional and practical value of digital and smart technologies in supporting student learning processes. 

Teacher digital literacy: One strategy identified in the literature is introducing preservice teacher training to improve technology recognition and confidence before teachers are introduced to these instruments in the classroom [23].

Student digital literacy: Student training and digital skills acquisition provide a foundation for using innovative applications and navigating new assistive technologies (e.g. visually impaired students using audio applications) productively in the classroom [18,21,22].

Discussion and analysis

Accessibility, QoL, and Student Needs

This study has explored an emergent trend in educational systems. The first research objective undertaken in this SLR was to define and critique the concept of accessibility in academic contexts and identify the emergent role of smart technologies in fulfilling these specific needs. The literature in this field showed that accessibility is defined differently according to circumstances and educational objectives. For example, Yang et al. (2023) detailed a politicized technological agenda in Chinese schools that viewed accessibility as a majority effect, with disparate learning experiences neglected in institutional policies and strategies [19]. Alternatively, a study described a form of technology-based scaffolding that integrates new, functional resources into student learning experiences according to skills development and functional proficiency. In the broader context of inclusive education, it was apparent from the SLR that accessibility in smart technologies represents both the availability and usability of advanced, integrated, and diversified resources for achieving positive, student-centered outcomes, including self-determination, autonomous learning, and classroom engagement [25].

The second research objective was to assess the particularities of educational inclusion from the perspective of special needs or disadvantaged learners. Within the SLR, it was evident that inclusion was a challenging condition of accessibility that required both teacher and student participation in a form of bottom-up integration. Harrison et al. (2020) demonstrated how cross-study meta-analysis of empirical evidence offers insights regarding the most positive and negative effects of accommodation on patient QoL [15]. Underlying variables, such as support levels, outreach, and self-determination, can then be used to operationalize intervention strategies that address systemic limitations or accommodation gaps that might directly improve long-term QoL [16]. When translated into school-based applications, the studies reviewed herein have confirmed that experimental methods, such as pre and post-intervention performance, can be used to weigh the effectiveness of technologies as learner supports and verification mechanisms for future program development.

In developing accessible learning systems, Vincent-Lancrin (2022) proposes that complementary but potentially competing needs profiles underlying teacher and student technological proficiency may threaten the efficacy of new, smart solutions [2]. Students might thrive using computers that feature recorded and direct, interactive lessons. Still, the teacher's ability to design and administer such applications is based upon specialized training and technological experiences. Similarly, where teachers might view advances in interactive lessons and online exercises as valuable, students’ technological proficiency levels will likely vary in educational settings. Such smart accessibility solutions may magnify systemic gaps and create barriers to learning for those students who lack the skills or capabilities needed to navigate these innovative and advanced resources [2]. Accordingly, researchers such as Starks and Reich (2023) and Loveys and Butler (2023) have proposed that special needs students not only require specific consideration in designing accessible technology solutions but should be integrated into the decision-making process as new technology benefits are weighed and objectified [17,22].

QoL and the Servitization of Accessible Smart Technologies

One of the core concepts identified when designing this study was the strong relationship between education as a service and student-centered accessibility effects on QoL-related outcomes. As evidenced by Subrahmanyam (2017), there is a strong correlation between service quality and recipient QoL contingent upon the affective value of service accommodation concerning the recipient’s needs, priorities, and outcomes or success [26]. As discussed by Gutierrez et al. (2023) in the SLR, without some form of performative benchmark or educational audit, the cross-student effectiveness of digital technologies, such as gamified lessons, remains incongruent [18]. Suppose students lack the digital literacy or confidence to engage with these games in productive ways or perhaps their learning needs diverge from the type of game (e.g. role-playing vs memorization). In that case, these effects may negatively impact the QoL of learners, particularly those with special needs [19].

The third research objective was to assess the relationship between accessibility and accommodation in recent changes to educational systems (e.g., hybrid learning) to identify opportunities for smart technology enhancement in the future. Customizing application interfaces for specific student needs represents a bridging solution that can include students with learning challenges or accessibility needs in the broader classroom environment [19,21]. For visually impaired students, software interfaces can magnify text, present visually tuned lessons, or incorporate auditory cues to resolve accessibility issues related to classroom handouts or blackboard teaching techniques. Ioana-Alexandra et al. (2021) demonstrate how smart speakers integrated with AI chatbots can supplement educational lectures and incorporate call-response queries into the full spectrum learning experience afforded to students with special needs like visual impairment or learning disabilities [9]. In their assessment of assistive technologies, Arslan-Ari and Baser (2023) demonstrated how enhanced and multi-platform training for special education teachers can positively impact classroom adaptations as student needs are identified and accommodated [23].

Despite the potential advantages of digitalization in educational settings, Yang et al. (2023) reported evidence regarding the exclusionary effects of data governance and digital biases in shaping modern educational practices [19]. Although often based on resource availability, the rush to integrate digital technologies and virtual learning solutions results in inequalities that exclude students from a learning arc that lacks evaluation and effect-weighted assessments [20]. Whereas such service level gaps could be eliminated through ecological assessments and individual student behavior monitoring, teachers lack the resources to perform these functions, and administrators are restricted to broad roles of decision-makers and technology-sourcing agents, not monitors. As predicted by the Buzo-Sanchez et al. (2023) SMART learning model, integrating technological resources can activate student self-determination by ensuring that accessible, adaptive, and meaningful technologies are integrated into daily learning activities [4].

Gaps, Problems, and Future Implications

Starks and Reich (2023) sampled teacher perspectives, highlighting the complex burden associated with pedagogy in a digital age but failed to consider the instrumental range of smart technologies that might be available for special needs applications and educational improvement [17]. As Kumm et al. (2021) demonstrated, when technology efficacy is oriented toward student awareness and self-determination, the productive value of such accessible solutions extends to QoL outcomes like self-determination and achievement [24].

One of the problems in defining QoL through academic research is that there is an assumption that exclusion or inaccessibility equates to declining QoL. For example, Starks and Reich (2023) inadvertently demonstrated that in some cases, QoL may increase for special needs students if they are excluded from new technologies because they avoid the literacy burdens and skills requirements that are being broadly imposed upon their peers [17]. In fact, throughout much of the research in this field, there was an assumed, negative correlation between exclusion from smart or advanced digital technologies and student learning opportunities that neglected many of the arguments posed by researchers. Connecting positive technological relationships to improved QoL over the educational lifecycle is a complex objective requiring additional longitudinal research, behavior tracking, and educational career mapping in future studies.

This study relies on purely secondary research structured and systematized through the formal procedures in this SLR to analyze a key problem in modern education. Although this approach has limited the potential for ethical complications, the selective nature of these keywords and this research procedure raise concerns about the reliability and validity of these findings. The adoption of the CASP (2023) assessment instrument was used to critically compare the overall value and validity of the studies included in this procedure [27]. By restricting the findings to empirical research that met these expectations, this investigation has eliminated many outlying opinions, problematic techniques, and unjustified conclusions [27]. Further, the generalizability of these findings, and therefore, the reliable extension of these insights to future problems or events, has been ensured by applying a comparative, thematic assessment to the cross-study evidence pool [28]. By triangulating the results of each study with prior literature and general conceptual foundations, the end result of this study was an academically justified, ethically responsible, and representative interpretation of educational accessibility, smart technologies, and student QoL.

## Conclusions

Incorporating novel technologies into modern educational systems has catalyzed a transformation toward digitally enhanced learning environments, offering unprecedented opportunities for personalized and interactive education. Addressing this digital divide requires inclusive, adaptable technologies alongside equitable implementation strategies. Studies emphasize that beyond providing technology, education systems must prioritize accessibility, teacher training, and policy support to maximize impact. By fostering inclusive learning environments and continuously assessing technological effectiveness, schools can enhance student engagement, well-being, and overall educational equity. Ensuring proactive measures will help bridge gaps and create a more inclusive and technology-driven future for all learners.

## Appendices

Appendix A 

**Table 3:** Search terms table

Type	Variations
Inclusive	Education AND Smart AND Technology AND Accessibility AND Quality of Life AND Special Needs AND Accommodation
Selective 1	Education AND Smart AND Accessibility OR Technology AND Self-Determination
Selective 2	Education AND Smart AND Accessibility OR Technology AND Quality of Life
Narrower, but Inclusive	Education AND Accessibility AND Smart AND Quality of Life OR Well Being

Appendix B 

**Table 4:** CASP appraisal checklist and data table CASP: Critical Appraisal Skills Programme

CASP Assessment Model (Adapted for both Quantitative and Qualitative Studies)												
			1	2	3	4	5	6	7	8	9	10
#	Author	Year	Was there a clear statement of the aims of the research?	Is the methodology appropriate?	Was the research design appropriate to address the aims of the research?	Was the recruitment strategy appropriate to the aims of the research?	Was the data collected in a way that addressed the research issue?	Has the relationship between researcher and participants been adequately considered?	Have ethical issues been taken into consideration?	Was data analysis sufficiently rigorous?	Is there a clear statement of findings?	How valuable is the research?
1	Gutierrez et al. [18]	2023	1	1	1	1	1	1	1	1	1	1
2	Yang et al. [19]	2023	1	1	2	1	1	1	1	1	1	1
3	Starks & Reich [16]	2023	1	2	1	1	1	1	1	1	1	1
4	Smith et al. [25]	2023	1	1	1	1	1	1	1	1	1	1
5	Arslan-Ari and Baser [23]	2023	1	1	1	1	1	1	1	2	1	1
6	Nordstrom et al. [20]	2019	1	2	1	1	2	1	1	1	1	2
7	Kumm et al. [24]	2021	1	1	1	1	1	1	1	1	1	1
8	Miguel-Revilla et al. [17]	2021	1	2	1	1	1	1	1	1	1	1
9	Loveys and Butler [21]	2023	1	1	1	1	1	1	1	2	1	1

Appendix C 

**Table 5:** SLR matrix table SLR: systematic literature review

SLR Output Matrix						
#	Author(s)	Year of Publication	Title	Methodology	Primary Finding	Significance
1	Gutierrez et al. [18]	2023	What do secondary teachers think about digital games for learning: Stupid fixation or the future of education?	Mixed methods research of teacher views regarding digital games and student learning applications in secondary education.	There is resistance to technological integration from experienced teachers who view such additional resources as displacements for contested space due to time and resource constraints. Although some teachers recognize digital learning as a form of social good, concerns regarding student addiction, particularly for special needs students could negatively impact QoL outcomes.	Despite their potential educational applications, in their current state, digital games lack the educational rigor and curricular auditing to meet the full spectrum needs of students. Disadvantages regarding digital literacy and/or digital dependency can create negative well-being impacts for students with learning disorders or special needs, reducing accessibility.
2	Yang et al. [19]	2023	Discourses regarding education governance in the digital age at K-12 Level: Possibilities, risks, and strategies.	Semi-structured interviews were conducted with 11 department heads and principals involved in front-line governance of Chinese K-12 schools in a single province.	Despite the integration of advanced technologies including AI, online monitoring platforms, and interactive applications, a framework of data determinism has restricted the inclusivity and universality of digitalization. Educational governance can lead to distorted representations of achievements due to the superficial use of data and a rising number of learning barriers or hurdles for some students.	To improve data governance and democratize a system based on the values and visions of a few individuals, more robust ecological assessments need to be conducted. Teacher and student surveys will assist in assessing the value of digitalization for students and improve inclusivity.
3	Starks & Reich [16]	2023	What about special ed? Barriers and enablers for teaching with technology in special education.	Multiple case study research with 20 semi-structured interviews designed to assess special educators' experiences with technology and teaching.	Despite the increasing availability of digital learning technologies, special education students are frequently left out of resource distribution, with SMART resources such as computers, AI devices, and interactive learning instruments distributed to general education classes first. Students often lack digital literacy support and training programs to improve resources. One-size-fits-all training does not address the range of special education needs and there is limited collaboration between special education and general education.	Many smart technologies are being purchased without input from teachers, parents, or students, resulting in multiple instruments, applications, and resources that lack practical applications in classroom settings. Families may lack digital literacy to support their students, and students may not have sufficient training or time to learn new skills. Special needs students often lack the support needed to develop skills, engage in classroom expectations, or use advanced technologies.
4	Smith et al. [25]	2023	A research agenda for computational empowerment for emerging technology education	Experimental design of participatory, SMART teaching technologies for realizing shared educational principles and learning outcomes.	Scaffolding strategies are developed to motivate student immersion in smart technologies, leveraging thinking platforms to construct inclusive solutions that avoid student marginalization. Student involvement encourages testing and role assessment through participatory design solutions.	Digital technologies such as facial recognition technology and smart sensors have the potential to enhance student engagement and active participation in lessons. However, due to traits and variables amongst users (e.g. visually impaired, non-mobile students, skills expectations), accessibility may be restricted. Practical testing and participatory design improve the accommodation of broader student inclusion and well-being.
5	Arslan-Ari and Baser [23]	2023	Assistive technology training within an educational technology course: Perceptions of preservice special education teachers	Survey of 67 PSETs before and after course completion to assess the effects of training on assistive technology competency and future classroom applications	Designing inclusive educational solutions requires the integration of teacher-specific knowledge and skill sets within a changing scope of student needs or applications. Educational training and real-world experimentation aid in developing awareness and improving mastery of higher technology skills.	Assistive technologies have a direct, positive effect on student learners with disabilities or special learning needs when used appropriately. Lack of training in new tools and technologies can restrict teacher effectiveness; thereby requiring support training and specialized preservice education.
6	Nordstrom et al. [20]	2019	Assistive technology applications for students with reading difficulties: Special education teachers' experiences and perceptions	A 6-week training intervention followed by a survey of 54 special education teachers' perceptions on how apps impacted student motivation and autonomy.	Assistive technologies that include various text-to-speech and speech-to-text functions can provide special education students with improved accessibility to classroom lessons. Where specific difficulties are identified, assistive technology can improve student motivation and engagement in the learning process.	The integration of functional applications with specific solutions for student learning is important to improving the overall benefit to the student body. Well-being for learners with reading or attention difficulties can be improved through application-based intervention using text or speech-based assistance. Customization would improve learner outcomes.
7	Kumm et al. [24]	2021	A technology-based self-monitoring intervention for secondary students with high-incidence disabilities	A two-teacher, 4-student practical experiment using self-monitoring software for students with IEPs and predetermined disabilities.	Collaborative technology allows teachers and students to monitor performance and behaviors in real-time to assist in refocusing or support. Self-monitoring resources increase student awareness regarding behavioral and attention deviations and allow for adaptations and improved classroom functioning.	The practical value of real-time monitoring technologies includes student-based well-being improvements related to motivation, focus, and engagement and teacher-based participation in active and time-based behavioral monitoring.
8	Miguel-Revilla et al. [17]	2021	Fostering engagement and historical understanding with a digital learning environment in secondary education.	A mixed methods study of student engagement with digital learning in a secondary classroom using 3 different experimental groups.	The integration of digital learning resources into hybridized classroom environments improves student engagement and increases positive learner outcomes relative to content engagement and self-directed learning.	These digital technologies are contingent upon user navigation and digital literacy. Further, student motivation and perceived value are important in stimulating future usage of technologies that supplement traditional learning environments.
9	Loveys and Butler [21]	2023	Teachers' and students' perspectives on the extent to which assistive technology maximizes independence	Experimental mixed methods study of teacher and student perceptions regarding assistive technologies in classroom settings	Assistive technologies are viewed as a form of support mechanism to improve student independence and facilitate productive learning outcomes that might otherwise be hindered by disabilities. Teachers are directly responsible for promoting the use of these technologies but often lack the knowledge or experience to recommend solutions for students.	When adopted by students with visual impairment, assistive technologies were found to have a positive impact on QoL, including autonomy and self-determination of learning pathways. Teaching staff lacked experience or knowledge in key technologies and reported a need for additional training and support.

Appendix D 

**Table 6:** Thematic analysis table

Thematic Analysis Table						
#	Author	Year	Core Themes	Impact Factors	Mediators	Outcome Profile
1	Gutierrez et al. [18]	2023	Digital Education, Gaming, Technology, Well-being, Impact, Pedagogy	Digital Experience, Learning Needs, Educational Efficacy, Teacher Attitudes, Student Confidence, Accessibility	Auditing, Purposing, Design, Focusing, Curriculum, Teachers, Administration, Student Direction	As educational aids, digital games have viability but require additional assessment in the future to justify their universal and regular applications in secondary education.
2	Yang et al. [19]	2023	Educational Governance, Digitalisation, Inclusion, Teachers, E-Learning, Personalisation	Administration, Curriculum, Digital Barriers, Accessibility, Skills, Knowledge	Oversight, Big Data, Assessment, Monitoring, Communication	Digital learning capabilities offer unique opportunities for inclusivity and e-learning support for students; however, administration and data governance are needed to democratize the services and solutions being deployed.
3	Starks & Reich [16]	2023	Special Education, Accessibility, Inclusion, Design, Structures, Administration, Participation	Skills, Experience, Time, Resources, Inclusion, Systems, Communication, Internet Connectivity, Planning	Teachers, Parents, Administrators, Applications, Students, Culture, Leadership	Despite a rising inventory of digital technologies, special needs students are being left out of educational planning, resulting in learning gaps and deficiencies in digital literacy that inhibit participation and restrict technology effectiveness.
4	Smith et al. [25]	2023	Digital Modalities, Learner Tools, Machine Learning, Facial Recognition, Technology Design, Participatory Design	Usage, Function, Inclusion, Accessibility, Data, Students, Academics, Operationalisation	Participation, Design, Pragmatism, Teachers, Students, Accessibility	Interdisciplinary, participatory design is needed to ensure accessibility and create valuable technologies with practical student applications that are inclusive and adaptive. Bottom-up solutions prioritize student learning over technological capabilities.
5	Arslan-Ari and Baser [23]	2023	Preparedness, Knowledge, Technology, Software, Awareness, Training, Exposure	Learning, Instruction, Support, Content, Practice, Engagement	Students, Teachers, Learning, Knowledge, Function, Materials, Resources, Awareness, Practice	Preservice teachers require specialized training with assistive technologies to improve competency in providing technology accessibility and functional value to special needs students.
6	Nordstrom et al. [19]	2019	Assistive, Technology, Applications, Resources, Teachers, Motivation, Engagement	Support, Personalisation, Specificity, Usage, Self-Direction, Appropriateness	Teachers, Students, Functions, Capabilities, Disabilities, Resources, Accessibility	Improvements to student learning experiences are positively associated with assistive applications that resolve specific, student-oriented difficulties (e.g. speech, listening, focus). Motivation and engagement are enhanced by app personalization.
7	Kumm et al. [24]	2021	Monitoring, Technology, Assessment, Behaviour, Self-Determination, Recognition, Adaptation	Participation, Coordination, Application, Student-Centred, Well-being, Motivation	Attention, Adaptability, Recognition, Teachers, Students, Support, Intervention	Self-determination is improved by recognizing behavioral or attention divergences and responding to them in the moment. Student QoL is improved through awareness monitoring and developmental advancement.
8	Miguel-Revilla et al. [17]	2021	Digital, Ecosystem, Learning, Self-Direction, Knowledge, Ecosystem, Detail, Focus	Student, Motivation, Engagement, Methods, Platform, Topic, Interactivity, Function, Performance	Teachers, Connection, Value, Effects, Accessibility, Resources, Focus, Motivation	Student engagement with digital learning is performance and utility-conditioned, resulting in varying interest levels and degrees of personal accountability.
9	Loveys and Butler [21]	2023	Assistive, Technology, Inclusion, Resources, Specifics, Students, Needs, Teacher	Awareness, Recognition, Functionality, Accessibility, Usage, Integration, Motivation	Independence, Training, Experience, Cost, Resources, Institution, Students	Students gain independence and self-determination from assistive technologies that can improve educational outcomes. Teachers are responsible for identifying and recommending technologies to administrators and parents.

## References

[1] Fűzi B, Géring Z, Szendrei-Pál E (2021). Changing expectations related to digitalisation and socialisation in higher education. Horizon scanning of pre- and post-COVID-19 discourses. Educ Rev.

[2] Vincent-Lancrin S (2022). Smart education technology: how it might transform teaching (and learning). New England Journal of Public Policy.

[3] Göksu H, Karanfiller T, Yurtkan K (2016). The application of smart devices in teaching students with special needs. The Turkish Online Journal of Educational Technology.

[4] Buzo-Sánchez I, Mínguez C, De Lázaro-Torres M (2023). The potential of the SMART learning framework to design and implement geospatial curricula in the secondary classroom. J Geogr.

[5] Van Hecke N, Claes C, Vanderplasschen W (2018). Conceptualisation and measurement of quality of life based on Schalock and Verdugo’s model: a cross-disciplinary review of the literature. Soc Indic Res.

[6] Brock ME, Schaefer JM, Seaman RL (2020). Self-determination and agency for all: supporting students with severe disabilities. Theory Pract.

[7] Guay F (2022). Applying self-determination theory to education: regulations types, psychological needs, and autonomy supporting behaviors. Can J Sch Psychol.

[8] Wehmeyer ML, Shogren KA, Palmer SB, Williams-Diehm KL, Little T, Boulton A (2012). Impact of the self-determined learning model of instruction on self-determination: a randomized-trial control group study. Except Child.

[9] Ioana-Alexandra T, Camelia Ş, Laura D (2021). Towards accessibility in education through smart speakers. An ontology based approach. Procedia Comput Sci.

[10] Lombardi M, Croce L, Claes C (2016). Factors predicting quality of life for people with intellectual disability: results from the ANFFAS study in Italy. J Intellect Dev Disabil.

[11] Verdugo MA, Aguayo V, Arias VB, García-Domínguez L (2020). A systematic review of the assessment of support needs in people with intellectual and developmental disabilities. Int J Environ Res Public Health.

[12] Morán L, Gómez LE, Verdugo MÁ, Schalock RL (2023). The quality of life supports model as a vehicle for implementing rights. Behav Sci (Basel).

[13] Erez AB, Kuhle S, McIsaac JL, Weintraub N (2020). School quality of life: cross-national comparison of students' perspectives. Work.

[14] Remington R (2020). A Step-by-Step Guide to Conducting an Integrative Review. https://link.springer.com/book/10.1007/978-3-030-37504-1.

[15] Harrison M, Singh Roy A, Hultqvist J (2020). Quality of life outcomes for people with serious mental illness living in supported accommodation: systematic review and meta-analysis. Soc Psychiatry Psychiatr Epidemiol.

[16] Starks AC, Reich SM (2023). “What about special ed?“: barriers and enablers for teaching with technology in special education. Comput Educ.

[17] Miguel-Revilla D, Calle-Carracedo M, Sánchez-Agustí M (2021). Fostering engagement and historical understanding with a digital learning environment in secondary education. E-Learning and Digital Media.

[18] Gutierrez A, Mills K, Scholes L (2023). What do secondary teachers think about digital games for learning: stupid fixation or the future of education?. Teach Teach Educ.

[19] Yang X, Zhu X, Chen D (2023). Discourses regarding education governance in the digital age at K-12 level: possibilities, risks, and strategies. Teach Teach Educ.

[20] Nordström T, Nilsson S, Gustafson S, Svensson I (2019). Assistive technology applications for students with reading difficulties: special education teachers' experiences and perceptions. Disabil Rehabil Assist Technol.

[21] Loveys M, Butler C (2023). Teachers’ and students’ perspectives on the extent to which assistive technology maximises independence. Br J Vis Impair.

[22] Faragher R, Van Ommen M (2017). Conceptualising educational quality of life to understand the school experiences of students with intellectual disability. J Policy Pract Intellect Disabil.

[23] Arslan-Ari I, Başer D (2023). Assistive technology training within an educational technology course: perceptions of preservice special education teachers. J Spec Educ Technol.

[24] Kumm S, Talbott E, Jolivette K (2021). A technology-based self-monitoring intervention for secondary students with high-incidence disabilities. J Spec Educ Technol.

[25] Smith E, Sumner P, Hedge C, Powell G (2023). Smart-speaker technology and intellectual disabilities: agency and wellbeing. Disabil Rehabil Assist Technol.

[26] Subrahmanyam A (2017). Relationship between service quality, satisfaction, motivation and loyalty: a multi-dimensional perspective. Qual Assur Educ.

[27] Purssell E, McCrae N (2020). How to perform a systematic literature review: a guide for healthcare researchers, practitioners and students. https://link.springer.com/book/10.1007/978-3-030-49672-2.

[28] Suri H (2020). Ethical considerations of conducting systematic reviews in educational research. Systematic Reviews in Educational Research.

